# Clinical and behavioral factors associated with undernutrition among highly active antiretroviral therapy users in Southwest Ethiopia

**DOI:** 10.3389/fnut.2022.914983

**Published:** 2022-12-22

**Authors:** Nigusie Shifera, Tewodros Yosef, Mengistu Mekonen

**Affiliations:** School of Public Health, College of Medicine and Health Sciences, Mizan-Tepi University, Mizan Teferi, Ethiopia

**Keywords:** highly active antiretroviral therapy (HAART), acquired immunodeficiency syndrome (HIV/AIDS), malnutrition, anti-retroviral therapy (ART), Ethiopia

## Abstract

**Background:**

Globally, about 1.9 billion adults are overweight or obese, while 462 million are underweight. These are primarily found in countries with low and middle incomes, such as Ethiopia. Undernutrition is a frequent health problem among people living with HIV/AIDS; however, no large-scale research, including several health facilities, has been conducted in Ethiopia. Thus, this study aimed to assess the nutritional status and nutrition-related factors among highly active antiretroviral therapy (HAART) users in public hospitals in Southwest Ethiopia.

**Methods:**

A cross-sectional facility study design was conducted in all public hospitals in Southwest Ethiopia from January to March 2021. A systematic sampling technique was used to select the study participants. The collected data were entered into EpiData 3.1 and then exported to SPSS version 24 for statistical analysis. Binary logistic regression analysis was done to identify the factors associated with the outcome variable. The level of significance was declared at a *P*-value of <0.05, with their corresponding 95% confidence level.

**Results:**

A total of 402 HAART users have participated with a 100% response rate. The proportion of undernutrition (BMI <18.5 kg/m^2^) and patients with overweight or obesity (BMI ≥25 kg/m^2^) were 29.3% [95% CI: (24.6–33.5)] and 10% [95% CI: (6.6–12.9)], respectively. Out of undernutrition patients, severe undernutrition (BMI <16 kg/m^2^) accounted for 5.6%. Factors, such as food insecurity [AOR: 3.21, 95% CI: (1.76–5.91)], history of diarrhea [AOR: 2.86, 95% CI: (1.96–6.78)], CD4 cell count ≤ [AOR: 4.72, 95% CI: (2.14–12.13)], and substance user [AOR: 4.12, 95% CI: (2.31–7.30)], were the independent factors of undernutrition.

**Conclusion:**

This study found that the prevalence of undernutrition was high compared with other settings. The government should also pay due attention to improving the treatment of HIV/AIDS by offering nutritional support services in hospitals. Moreover, policymakers and healthcare professionals consider the effects of these factors on nutrition while providing ART services.

## Introduction

There are over 38 million patients with HIV/AIDS in the world, with over 25.5 million individuals living in Africa. Similar to this, while 12.6 million individuals are still waiting for highly active antiretroviral therapy (HAART), about 25.4 million people are being served (1). Despite 1.7 million new infections and 690,000 AIDS-related deaths recorded in 2019, 0.7% of those infections and fatalities were people aged 15–49 years (1, 2). According to EDHS research in Ethiopia, prevalence among adults was just nearly 1% in 2016 (3).

Undernutrition is caused by a lack of nutrients and energy to meet the body’s requirements for growth and maintenance (4). Globally, 462 million individuals are underweight, whereas over 1.9 billion are overweight or obese, and these mainly occur in countries with low and medium incomes (4). Ethiopia is among the largest incidences of undernutrition in the world, with almost 49% of the population lacking adequate nutrition (5).

Acquired immunodeficiency syndrome and human nutrition are tightly related, and each of them has the potential to gradually harm the immune system. Malnutrition makes matters worse by hastening the transition from HIV infection to AIDS. HIV/AIDS is frequently linked to biological and societal variables that impair people’s capacity to consume and utilize food (6).

Patients with HIV/AIDS might suffer from malnutrition for a variety of reasons. Food consumption disorder, nausea, and vomiting-inducing drugs, anorexia, opportunistic infections, diarrhea, nutritional malabsorption, and wasting syndrome are some of these reasons (7). Furthermore, HIV has a specific impact on the nutritional status by raising energy needs due to alterations in metabolism and oxidative processes. Compared with healthy persons, adult patients with HIV/AIDS need 10% more energy when they are asymptomatic, 20–30% more when they are symptomatic, and 30% more energy while recovering (8).

Studies revealed that adult HAART users frequently experience undernutrition to varying degrees. For example, studies conducted in Zambia and Cameroon revealed that the prevalence of undernutrition was 36.4 and 19.34%, respectively (9, 10). Various studies in Ethiopia have found varying degrees of malnutrition among HIV-positive adults. For instance, a study in the West Shewa public health faciliy 23.6% (11), in the East Harerge zone demonstrated that 30% (12), in Jimma Medical Center 34% (13), in Jimma town Oromia region 43% (14) of patients with HIV positive had malnutrition.

Even though many studies have been conducted to investigate undernutrition among adult HIV-positive individuals (11–17), no study has been conducted on large scale, incorporating several health facilities covering large demographic areas and sociocultural diversity. The results of this study will be helpful as input for the regional governmental and non-governmental groups who operate in the areas of HIV/AIDS and undernutrition. Therefore, this study aimed to assess the nutritional status and nutrition-related factors among HAART users in public hospitals in Southwest Ethiopia.

## Materials and methods

### Study setting, design, and period

From 1 January to 30 March 2021, a cross-sectional institutional study was carried out in public hospitals in the three zones of Southwest Ethiopia. Six hospitals, Wacha Maji Primary Hospital, Tepi Primary Hospital, Mizan-Tepi University Teaching Hospital, Gebretsadik Shawo General Hospital, Siz Primary Hospital, and Bonga Primary Hospital, are located in the three zones of Southwest Ethiopia. Three hospitals—Wacha Maji, Gebretsadik Shawo, and Mizan-Tepi University teaching hospitals—were chosen at random from a total of six public hospitals in the area. Maize, coffee, godere (taro root), and ensete are the principal food crops widely grown in the southwest region. The three public hospitals had an average of 5,215 patients with HIV positive on HAART, with 3,120 men and 2,095 women.

### Populations

All adult patients with HIV positive/AIDS who were receiving HAART at public hospitals in Southwest Ethiopia comprised the source population. The study population consisted of all adult patients with HIV/AIDS who were receiving HAART at public hospitals in Southwest Ethiopia and who met the eligibility requirements. All patients with HIV positive who are 18 years of age or older and receiving HAART at public hospitals in Southwest Ethiopia were included in the study. A person who was critically ill had hearing loss and a severe mental disorder at the time of the trial was omitted.

### Sample size determination and sampling technique

A single population proportion formula was used to calculate the sample size with a 95% confidence level, a 5% margin of error, and considering 5% non-response rate. The estimated prevalence of undernutrition among HAART users in Southwest Ethiopia was used (46.8%) (16). As a result, the final sample size became 402.

According to the total number of HAART clients in each public hospital, a proportionate number of samples were assigned for each HAART clinic. A systematic sampling technique was employed to select the study participants. We calculated the skip interval (*k* = 13). The random start was selected using a lottery method; then, the other study participants were selected every 13 intervals.

### Variables

Undernutrition was the dependent variable, and sociodemographic factors (sex, age, residence, religion, educational level, occupation, and income), clinical factors (CD4 count, viral load, WHO staging, diarrhea, HAART duration, TB treatment, and hemoglobin level), behavioral factors (substance use, alcohol use, nutritional counseling, and extra sexual partner), and vulnerability characteristics (household food consumption and food aid) were the independent factors.

### Measurements and operational definitions

#### Underweight, normal weight, overweight, and obese

The person who has a body mass index (BMI) of 18.49 kg/m^2^, less than, 18.5 to 24.9 kg/m^2^, greater than 25 kg/m^2^, and greater than 30 kg/m^2^ (18).

#### Household food secure

If the head of the household answers “no” to all of the questions listed from 1 to 9 or “yes” to question item 1 but says “rarely” in the last 4 weeks (19).

#### Household food insecure

If household heads affirmatively answer at least yes to question item 1 and have occasionally experienced this in the last 4 weeks (19).

#### Current substance use

According to the Alcohol, Smoking, and Substance Involvement Screening Tool (ASSIST), using at least one of a particular substance (alcohol, khat, cigarette, and others) for non-medical purposes for the last 3 months (20).

### Data collection procedures and tools

A structured interviewer-administered questionnaire, checklist, and measurements were used to collect the data. As data collectors and supervisors, three nurses and one public health expert, respectively, were hired. Sociodemographic information, clinical variables, and household and behavioral patterns were all included in the questionnaires.

Anthropometric assessments using body mass index were used to determine the nutritional status of the study subjects (BMI). A beam balance with a measurement was used to calculate the study participants’ weight, and a vertical height scale was used to measure the respondents’ heights. The BMI was then computed by multiplying the weight in kilograms by the square of the height in meters (kg/m^2^). As a result, study participants were classified as undernourished if their BMI was less than 18.5 kg/m^2^ (underweight) (21).

A validated Household Food Insecurity Access Scale (HFIAS) was used to evaluate the state of household food security (19), and ASSIST was used to assess the current substance use history of the participants (20).

### Data quality assurance

The English version of the questionnaire was translated into the local language Amharic and back-translated into English to ensure its consistency. A pretest was done among 5% of the study sample size. The training was given to the data collector and supervisor on the study objective, ethical standards, and data collection technique. Data were checked for completeness before being entered into EpiData 3.1. Descriptive analysis was used to check missing values and outliers before regression analysis.

### Data processing and analysis

The cleaned data were entered into EpiData 3.1 and exported to SPSS version 22 for analysis. Frequency, tables, and graphs were used to present the descriptive statistics. The assumption of normality was examined for continuous variables. The dependent variable (BMI) was determined by dividing the weight in kilograms by the square of the height in meters. Undernutrition was defined as having a BMI of less than 18.5 kg/m^2^ (15). Variables with a *P*-value of <0.25 were taken into multiple logistic regression analysis to identify independent predictors of undernutrition.

A model was built with a backward likelihood ratio with a 0.1 probability of removal. The final model’s goodness of fit was examined using the Hosmer–Lemeshow test of goodness of fit, which considers good fit at *P*-values of >0.05. At a cutoff *P*-value of 0.05, independent predictors of undernutrition were finally identified. The strength of the link was then evaluated using AORs and their related 95% confidence intervals.

### Ethics statement

The research was approved by the Institutional Review Board (IRB) of Mizan-Tepi University (Ref. No. MTU/20/45/8/35/11). Letters of approval were taken from each hospital. A brief explanation of the study’s objective and purpose was given to each participant, confidentiality was upheld, and written informed consent was obtained from each participant.

## Results

### Sociodemographic characteristics

A total of 402 adult HAART users participated with a 100% response rate. The mean BMI (SD) and age of the participants were 20.2 (2.9) kg/m^2^ and 34.1 (11) years, respectively. Women in the age group of 25–34 years were equally affected by both undernutrition and overweight. Of the 118 respondents with undernutrition, 66 (55.9%) and 49 (41.5%) of the respondents were women and in the age group of 25–34 years, respectively. Among overweight respondents, 22 (55%) and 16 (42.1%) of the respondents were women and in the age group of 25–34 years, respectively. Of the 118 respondents with undernutrition, 74 (64.7%) and 67 (56.7%) of the respondents were married and urban residents, respectively. Of the 118 respondents with undernutrition, 45 (38.1%) of the respondents achieved primary education (Table 1).

**TABLE 1:** Sociodemographic characteristics of adult HAART users grouped by nutritional status (BMI) at public hospitals in Southwest Ethiopia, 2020 (*N* = 402).

Variables	Undernutrition no. (%)	Normal weight no. (%)	Overweight/above no. (%)	Total no. (%)
**Sex**				
Male	52 (44.1)	116 (47.5)	18 (45.0)	186 (46.3)
Female	66 (55.9)	128 (52.5)	22 (55.0)	216 (53.7)
**Age**				
18–24	24 (20.3)	33 (13.5)	10 (25.0)	67 (16.6)
25–34	49 (41.5)	116 (47.5)	16 (42.1)	181 (45.0)
35–44	25 (21.2)	50 (20.5)	6 (15.8)	81 (20.2)
≥45	20 (16.9)	45 (18.4)	8 (21.1)	73 (18.2)
**Residence**				
Urban	67 (56.7)	146 (59.8)	29 (72.5)	242 (60.2)
Rural	51 (43.2)	98 (40.2)	11 (27.7)	160 (39.8)
**Religion**				
Orthodox	53 (47.4)	92 (37.7)	20 (50.0)	165 (41.0)
Muslim	21 (17.8)	75 (30.8)	10 (25.5)	106 (26.4)
Protestant	44 (37.2)	77 (31.5	10 (25.5)	131 (33.6)
**Marital status**				
Single	16 (13.5)	34 (13.9)	12 (30.0)	62 (15.4)
Married	74 (62.7)	153 (62.7)	21 (52.5)	248 (61.7)
Widowed	15 (12.7)	35 (14.3)	6 (15.0)	56 (13.9)
Divorced	13 (11.1)	22 (9.1)	1 (2.5)	36 (8.9)
**Educational level**				
Uneducated	28 (23.7)	23 (9.4)	2 (5.0)	53 (13.2)
Primary	45 (38.1)	84 (34.4)	18 (45.0)	147 (36.6)
Secondary	33 (27.9)	101 (41.4)	11 (27.5)	145 (36.1)
Tertiary	12 (10.2)	36 (14.7)	9 (22.5)	57 (14.1)
**Occupation**				
Unemployed	25 (21.1)	38 (15.6)	6 (15.0)	69 (17.2)
Housewife	16 (13.6)	29 (11.8)	5 (12.5)	50 (12.5)
Daily labor	16 (13.6)	23 (9.4)	5 (12.5)	44 (10.9)
Merchant	37 (31.4)	100 (41.0)	14 (35.0)	151 (37.5)
Government employee	24 (20.3)	54 (22.1)	10 (25.0)	88 (21.9)
**Average monthly income**				
≤2,500 ETB	70 (59.3)	104 (42.6)	16 (40.0)	190 (47.3)
>2,500 ETB	48 (40.1)	140 (53.4)	24 (60.0)	212 (52.7)

### Clinical-related characteristics

Of the 118 respondents with undernutrition, 50 (42.4%) and 80 (67.8%) of the respondents took HAART for more than 12 months and had a history of hospital admission, respectively. However, of the 30 respondents with overweight, 15 (37.5%) and 24 (60%) of the respondents took HAART for more than 12 months and had a history of hospital admission, respectively. Of the 118 respondents with undernutrition, 72 (61%) and 46 (39) of the respondents were in the WHO sage II and with a CD4 count of 200–500, respectively. However, of the 30 respondents with overweight, 29 (72.5%) and 15 (37.5%) of the respondents were in the WHO sage II and with a CD4 count of 200–500, respectively (Table 2).

**TABLE 2:** Clinical- and lifestyle-related characteristics and nutritional status of adults receiving HAART at public hospitals, Southwest Ethiopia, 2020.

Variables	Undernutrition no. (%)	Normal weight no. (%)	Overweight or above no. (%)	Total no. (%)
**HIV diagnosis**				
≤2 years	50 (42.4)	97 (39.7)	24 (60.0)	171 (42.5)
>2 years	68 (57.6)	147 (60.3)	16 (40.0)	231 (57.5)
**HAART duration (months)**				
<6 months	31 (26.3)	57 (23.4)	11 (27.5)	99 (24.6)
6–12 months	37 (31.3)	87 (35.6)	14 (35.0)	138 (34.3)
>12 months	50 (42.4)	100 (41.0)	15 (37.5)	165 (41.1)
**CD4 count**				
≤200	35 (29.6)	19 (7.8)	6 (15.0)	60 (15.0)
200–500	46 (39.0)	126 (51.6)	19 (47.5)	191 (47.5)
=500	37 (31.4)	99 (40.6)	15 (37.5)	151 (37.5)
**WHO staging**				
Stage I	11 (9.3)	37 (15.2)	5 (12.5)	53 (13.2)
Stage II	72 (61.0)	175 (71.7)	29 (72.5)	276 (68.6)
Stage III	22 (18.6)	20 (8.2)	2 (5.0)	44 (11.0)
Stage IV	13 (11.1)	11 (4.5)	4 (10.0)	29 (7.2)
**Hemoglobin level**				
≤12 mg/dl	56 (47.4)	79 (32.4)	6 (15.0)	141 (35.1)
>12 mg/dl	62 (52.6)	165 (67.6)	34 (85.0)	261 (64.9)
**Diarrhea**				
Yes	55 (46.6)	55 (22.5)	13 (32.5)	123 (30.6)
No	63 (53.4)	189 (77.5)	27 (67.5)	279 (69.4)
**TB**				
Yes	34 (28.8)	37 (15.2)	6 (15.0)	77 (19.2)
No	84 (71.2)	207 (84.8)	34 (85.0)	325 (81.8)
**Hospital admission**				
Yes	80 (67.8)	128 (52.4)	24 (60.0)	232 (57.7)
No	38 (32.2)	116 (47.6)	16 (40)	170 (42.3)
**Opportunistic infection**				
Yes	40 (33.0)	37 (15.2)	7 (17.5)	84 (21.0)
No	78 (67.0)	207 (84.8)	33 (82.5)	318 (79.0)
**Difficulty of swallowing**				
Yes	15 (12.7)	24 (9.8)	1 (2.5)	40 (10.0)
No	103 (87.3)	220 (90.2)	39 (97.5)	362 (90.0)
**Comorbidity**				
Yes	37 (31.6)	46 (18.8)	7 (17.5)	90 (22.4)
No	81 (69.4)	198 (81.2)	33 (82.5)	304 (77.6)
**Food aid**				
Yes	38 (32.2)	71 (29.1)	14 (35.0)	123 (30.6)
No	80 (67.8)	173 (70.9)	26 (65.0)	279 (69.4)
**Food insecurity**				
Yes	28 (23.7)	127 (52.0)	19 (47.5)	174 (43.3)
No	90 (77.3)	117 (48.0)	21 (52.5)	228 (56.7)

### Behavioral-related characteristics

This study also addressed the behavioral characteristics of the participants. The majority of people who were malnourished had histories of HIV disclosure and substance use, with 93 (70.8%) and 56 (47.4%), respectively. However, among people who were overweight, around one-fourth (25%) used substances (Table 3).

**TABLE 3:** Behavioral characteristics and nutritional status of adults receiving HAART at public hospitals in Southwest Ethiopia, 2020.

Variables	Undernutrition no. (%)	Normal weight no. (%)	Overweight or above no. (%)	Total no. (%)
**HIV disclosure**				
Yes	93 (78.8)	186 (76.2)	36 (90.0)	315 (78.3)
No	25 (21.2)	58 (23.8)	4 (10.0)	79 (21.7)
**Nutritional counseling’s**				
Yes	75 (63.5)	143 (58.6)	23 (57.5)	241 (59.9)
No	43 (36.5)	101 (41.4)	17 (42.5)	161 (41.1)
**Current substance use**				
Yes	56 (47.4)	46 (18.8)	10 (25.0)	111 (27.4)
No	62 (52.6)	198 (81.2)	30 (75.0)	290 (72.6)
**Extramarital sex**				
Yes	44 (37.3)	108 (44.3)	15 (37.5)	167 (41.5)
No	74 (62.7)	136 (55.7)	25 (62.5)	235 (58.5)

### Prevalence of malnutrition

**FIGURE 1:**
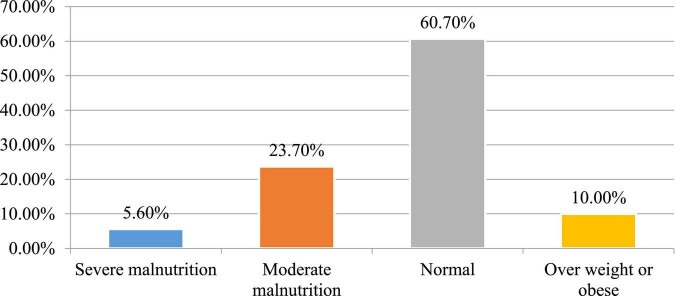
Nutritional status of adult HAART users in public hospitals in Southwest Ethiopia, 2020.

The proportion of patients with undernutrition (BMI <18.5 kg/m^2^), normal nutrition (BMI 18.5–24.9 kg/m^2^), and overweight or obese (BMI ≥25 kg/m^2^) were 29.3% [95% CI:(24.6–33.5)], 60.7% [95% CI: (56.5–66.3)], and 10% [95% CI: (6.6–12.9)], respectively. Out of patients with undernutrition, severe malnutrition (BMI <16 kg/m^2^) accounted for 5.6% (Figure 1).

### Predictors of undernutrition

From the total 11 candidate variables entered into multivariable analysis, four variables were found to be independently associated with undernutrition among adult patients with HIV positive attending HAART. Undernutrition was about three times [AOR: 3.21, 95% CI: (1.76–5.91)] more likely among patients who had food insecurity than those who had not.

The odds of undernutrition were four times [AOR: 2.86, 95% CI: (1.96–6.78)] higher among patients with HIV who had a history of diarrhea. Patients with HIV/AIDS and CD4 cell count of ≤200 cells/mm^3^ were nearly six times [AOR: 4.72, 95% CI: (2.14–12.13)] more likely undernourished than patients with a CD4 cell count of ≥500 cells/mm^3^. Moreover, individuals who were substance users are about four times [AOR: 4.12, 95% CI: (2.31–7.30)], a higher risk of developing undernutrition as compared to those who did not use it (refer to Table 4).

**TABLE 4:** Multivariable analysis of different variables with undernutrition among adult HAART users at public hospitals in Southwest Ethiopia, 2020.

Variables	Undernutrition no. (%)	Normal nutrition no. (%)	COR (95% CI)	AOR (95% CI)	*P*-value
**Monthly income**					
≤50 USD	70 (59.3)	104 (42.6)	1.96 (1.30–3.22)	1.63 (1.00–2.98)	0.067
>50 USD	48 (40.1)	140 (53.4)	1		
**CD4 count**					
≤200	35 (29.6)	19 (7.8)	4.93 (2.43–10.43)	4.72 (2.14–12.13)	<0.0001*
200–500	46 (39.0)	126 (51.6)	0.98 (0.51–1.73)	0.74 (0.39–1.36)	0.331
=500	37 (31.4)	99 (40.6)	1	1	
**WHO staging**					
Stage I	11 (9.3)	37 (15.2)	1	1	
Stage II	72 (61.0)	175 (71.7)	1.38 (0.67–2.86)	1.58 (0.60–4.14)	0.348
Stage III	22 (18.6)	20 (8.2)	3.70 (1.50–9.15)	1.17 (0.36–3.81)	0.793
Stage IV	13 (11.1)	11 (4.5)	3.97 (1.42–11.62)	2.62 (0.77–11.65)	0.123
**Hemoglobin level**					
≤12 mg/dl	56 (47.4)	79 (32.4)	1.80 (1.21–3.22)	1.67 (0.95–3.20)	0.61
>12 mg/dl	62 (52.6)	165 (67.6)	1	1	
**Food insecurity**					
Yes	28 (23.7)	127 (52.0)	3.50 (2.13– 6.78)	3.21 (1.76–5.91)	<0.0001*
No	90 (77.3)	117 (48.0)	1	1	
**Diarrhea**					
Yes	55 (47.8)	52 (21.6)	3.33 (2.07–5.37)	2.86 (1.96 –6.78)	0.003*
No	60 (52.2)	189 (78.4)	1	1	
**TB**					
Yes	34 (28.8)	37 (15.2)	2.26 (1.30–3.89)	1.35 (0.61–2.99)	0.457
No	84 (71.2)	207 (84.8)	1	1	
**Hospital admission**					
Yes	80 (67.8)	128 (52.4)	1.91 (1.24–3.21)	1.47 (0.85–2.61)	0.160
No	38 (32.2)	116 (47.6)	1	1	
**Opportunistic infection**					
Yes	40 (33.0)	37 (15.2)	2.86 (1.64–4.48)	1.12 (0.44–2.25)	0.999
No	78 (67.0)	207 (84.8)	1	1	
**Comorbidity**					
Yes	37 (31.6)	46 (18.8)	1.97 (1.32–3.44)	0.74 (0.31–1.73)	0.435
No	81 (69.4)	198 (81.2)	1	1	
**Current substance use**					
Yes	56 (47.4)	46 (18.8)	3.90 (2.50–6.46)	4.12 (2.31–7.30	<0.0001*
No	62 (52.6)	198 (81.2)	1	1	

*p < 0.05.

## Discussion

The prevalence of undernutrition and overweight or obesity was 29.3 and 9.6%, respectively. Out of patients with undernutrition, severe undernutrition (BMI <16 kg/m^2^) accounted for 5.6%. Moreover, food insecurity, history of diarrhea, CD4 count, and current substance user were the independent factors associated with undernutrition among adult HAART users.

The prevalence of undernutrition was close to that of a study conducted in various parts of Ethiopia: 30% in hospitals in the East Harerge zone (12), 25.2% in Butajira Hospital (22), and 27% in Nekemte Referral Hospital (23); however, the prevalence of this study was lower than 60.2% in west Gojam zone public hospitals (17), 46.8% in Jimma University specialized hospital, (16) 43% in Southwest Oromia region (14), and higher than study conducted in 12.3% in Dilla University Hospital (24) and 18.2% in Arba Minch area public health facilities (15). Similarly, the prevalence is also much higher compared to studies done in different parts of the world: 19.5% in Tanzania (25), 10% in Zimbabwe (9), and 19.2% in Senegal (26). The potential difference in the prevalence of malnutrition could be attributable to socioeconomic, food, and cultural factors.

Food insecurity is significantly associated with undernutrition. Patients with HIV who had food insecurity were 3.2 times more likely undernourished when compared with their counterparts. Similarly, the research done in the East Hararge Zone hospitals of Ethiopia and Senegal found that patients with household food deficiency had higher risks of undernourishment (12, 26). This is because there is insufficient food to satisfy nutritional requirements for productive and healthy living, contributing to macronutrient and micronutrient deficiencies.

There was a strong correlation between CD4 counts and undernutrition. Patients with a CD4 count of <200 cells/mm^3^ were approximately 4.7 times more likely to grow under undernutrition than those with a CD4 count of >500 cells/mm^3^ to grow under undernutrition. Similarly, in Jimma, Ethiopia, Senegal, and Tanzania, the study found that CD4 was significantly associated with lower CD4 counts (13, 25, 26). On the other hand, the results of research conducted in the southern part of Ethiopia at Dilla University Hospital indicate that CD4 has no major impact on nutrition (24). Contrary to our research results, the disparity could be due to differences in the cultural variation of the study population and the study duration (12).

Patients with HIV and diarrhea were nearly three times more likely to develop undernutrition compared with patients with HIV and without diarrhea. Since HIV/AIDS can cause undernutrition directly and also indirectly through opportunistic infections, infectious diarrhea is the commonest opportunistic infection linked to undernutrition in HIV/AIDS (21). This finding was consistent with previous studies conducted in Ethiopia (12, 27, 28). This is because diarrhea increases the risk of undernutrition by reducing food appetite, energy intake, increasing nutrient loss, and decreasing nutritional absorption (29).

Current substance use has a significant effect on undernutrition. Patients who used any substance in the last 3 months were four times more likely to develop undernutrition as compared to those who had not used it. A study done in Arbaminch and Canada showed that current tobacco-smoking adults enrolled in HAART programs were at a higher risk of being undernourished (15, 30). However, a study in Nepal showed behavioral factors such as alcohol and smoking were found to have no association with undernutrition (31). The possible difference may be due to differences in the study population, study design, and period. Therefore, Ethiopia’s nutritional strategy needs to take into account the role that substance abuse plays in patients with HIV and undernutrition.

## Conclusion and recommendation

This study revealed that the prevalence of undernutrition was high compared with other settings in Ethiopia. It was also shown that food insecurity, history of diarrhea, CD4 cell count ≤200 cells/mm^3^, and substance use were independently associated with undernutrition among adult HAART users.

This demands the government give due attention to strengthening HIV/AIDS treatment, nutritional assessment, supplementation, counseling, care, and support to patients and support services at hospitals. In addition, the implications of food insecurity, diarrheal, CD4 cell count of fewer than 200 cells/mm^3^, and substance use should be considered by policymakers and health professionals working at the HAART clinic on the nutritional status among adult patients with HIV.

### Limitations of the study

Since the data are cross-sectional, it is challenging to determine how the study variables are related to one another.

## Data availability statement

The raw data supporting the conclusions of this article will be made available by the authors, without undue reservation.

## Ethics statement

The studies involving human participants were reviewed and approved by the Mizan-Tepi University. The patients/participants provided their written informed consent to participate in this study.

## Author contributions

NS, TY, and MM wrote the protocol, participated in data collection, analyzed the data, and wrote the manuscript. All authors read and approved the final manuscript.
